# Prognostic Role of MicroRNA-126 for Survival in Malignant Tumors: A Systematic Review and Meta-Analysis

**DOI:** 10.1155/2015/739469

**Published:** 2015-08-17

**Authors:** Jie Bu, Hui Li, Xiao-yang Li, Li-hong Liu, Wei Sun, Tao Xiao

**Affiliations:** Department of Orthopedics, The Second Xiangya Hospital of Central South University, Changsha 410010, China

## Abstract

*Background.* Increasing studies found that miR-126 expression may be associated with the prognosis of cancers. Here, we performed a meta-analysis to assess the prognostic role of miR-126 in different cancers. *Methods.* Eligible studies were identified by searching in PubMed, Embase, the Cochrane Library, CNKI, and Wan Fang databases up to March 2015. Pooled hazard ratios (HRs) and their corresponding 95% confidence intervals (CIs) were calculated to investigate the correlation between miR-126 and survival of cancers. *Results.* Thirty studies including a total of 4497 participants were enrolled in this meta-analysis. The pooled results showed that high level of miR-126 was a predictor for favorable survival of carcinomas, with pooled HR of 0.77 (95% CI 0.64–0.93) for OS, 0.64 (95%CI 0.48–0.85) for DFS, and 0.70 (95% CI 0.50–0.98) for PFS/RFS/DSS. However, high level of circulating miR-126 predicted a significantly worse OS in patients with cancer (HR = 1.65, 95% CI 1.09–2.51). *Conclusions.* Our results indicated that miR-126 could act as a significant biomarker in the prognosis of various cancers.

## 1. Introduction

MicroRNAs (miRNAs), which are a new class of small noncoding RNAs (21–23 nucleotides), have emerged as crucial players regulating the magnitude of gene expression in a variety of organisms [1, 2]. Regulation of microRNAs is achieved via binding to the 3′ untranslated regions (3′ UTR) of target mRNAs, which leads to their inhibition of the expression of target genes in the translation level [3]. Mounting evidence suggests that microRNAs play crucial and complex roles in the initiation and progression of cancer [4], including cell proliferation, differentiation, apoptosis, and metabolism [5, 6]. Obviously, microRNAs may be exploited as new promising molecular biomarkers for early diagnosis and efficient treatment in human cancers [7].

MicroRNA-126 (miR-126), located within the 7th intron of* EGFL7* (epidermal growth factor-like domain 7), plays an important role in cellular biology, including cancer biology [8, 9]. Many studies have demonstrated that miR-126 contributes to progression of angiogenesis, proliferation, migration, invasion, and cell survival in some cancers [8, 10–12]. As a tumor suppressor, miR-126 was shown to downregulate expression in lung, breast, gastric, colon, pancreatic, oral, and some other cancers in previous studies [13–18]. Cancer patients with lower expression of miR-126 always had a worse prognostic outcome; however, the results from different studies indicated that miR-126 functioned as an oncogene and its expression was upregulated [19–22].

The majority of cancers at the time of initial diagnosis are often at an advanced stage and have poor prognosis, and therefore there is an urgent need for the identification of novel prognostic and predictive biomarkers to improve treatment of patients with various cancers [23]. In spite of some contradictory results, miR-126 is still a significant tumor biomarker and a potential therapeutic target [24]. Moreover, the result from individual study is inadequate to evaluate whether miR-126 can be considered as a promising biomarker. So we performed this meta-analysis to assess the prognostic value of tissue and blood-based miR-126 levels in various cancers.

## 2. Materials and Methods

This meta-analysis was performed following the guidelines of the Systematic Reviews and Meta-Analyses (PRISMA) and the Observational Studies in Epidemiology group (MOOSE) [25].

### 2.1. Search Strategy

Literatures were systematically searched through PubMed, Embase, the Cochrane Library, CNKI (China National Knowledge Infrastructure), and Wan Fang databases up to March 2015 without any language restrictions by two independent reviewers (Jie Bu and Hui Li). The search strategy of key words and their combination was the following terms: “microRNA-126 OR miR-126 OR miR-126-3p” AND “tumor OR tumour OR neoplasm OR cancer OR carcinoma” AND “prognosis OR survival OR outcome OR prognostic.” We also carefully performed a manual search in order to identify other potentially eligible studies.

### 2.2. Inclusion and Exclusion Criteria

The eligible studies in this systematic review must meet all the following criteria: (1) patients are included with any type of cancers, (2) the association between miR-126 expression and survival outcome was measured in cancerous tissues or circulatory system, and (3) sufficient data was provided to calculate the hazard ratio (HR) and 95% confidence intervals (CIs). Articles were excluded according to the following criteria: (1) letters, case reports, reviews, conference abstracts, and animal or laboratory studies, (2) studies analyzing a set of miRNAs altogether and nondichotomous miR-126 expression levels, and (3) studies with fewer than 30 patients. When the same patient cohort was reported from multiple published data, only the most recent or complete study was selected.

### 2.3. Quality Assessment and Data Extraction

Quality assessment of included studies was assessed by two researchers independently (Jie Bu and Hui Li) following a critical review checklist of the Dutch Cochrane Centre proposed by MOOSE [25]. The following items were included: first author's name, publication year, country or area of origin, cancer type, sample type, TNM stage, method, total number of patients, cut-off value, follow-ups and HRs of miR-126 for overall survival (OS), disease-free survival (DFS), recurrence-free survival (RFS), progression-free survival (PFS), and disease-specific survival (DSS), with their 95% confidence intervals (CIs). Disagreements were resolved by discussion between these reviewers (Jie Bu, Hui Li, and Xiao-yang Li) or consultation with senior reviewer (Li-hong Liu). If both univariate and multivariate analysis results were reported for survival, the latter ones would be selected [26, 27].

We extracted the statistical variables according to the following methods. If HRs and 95% CIs were described in publications, we extracted them directly. Otherwise, survivals and deaths at specified times in each group were extracted to calculate HRs. If only Kaplan-Meier curves are available, they were extracted from the graphical survival plots to estimate the HRs following the previously described method [28, 29]. We used Engauge Digitizer version 4.1 to extract the data from Kaplan-Meier survival curves, and three independent researchers (Jie Bu, Hui Li, and Xiao-yang Li) read the curves to reduce reading variability. We also contacted the authors of eligible articles by email for additional information and the essential data needed for the meta-analytic calculations.

### 2.4. Statistical Analysis

HRs with their 95% CIs were combined to evaluated the effect of miR-126 expression on the survival outcome of cancer. Patients with overexpression of miR-126 indicated a better prognosis if HR < 1 and its 95% CI did not overlap with 1. Heterogeneity of pooled HRs was carried out using Cochran's *Q*-test and Higgins *I*-square (*I* ^2^) statistic [30, 31]. If there was significant heterogeneity (*P* < 0.05 or *I* ^2^ > 50%.), the random-effects model (Der Simonian and Laird method) was used [32]. Otherwise, a fixed-effects model (Mantel-Haenszel test) was applied [33]. Subgroup analysis and metaregression were further performed to explore possible explanations for heterogeneity. Begg's funnel plot and Egger's bias were used to evaluate the potential publication bias [34, 35]. Analysis of sensitivity was performed to evaluate the stability of the results. All statistical tests were two-sided, and *P* < 0.05 was regarded as statistically significant. All analyses were conducted using the Cochrane Collaboration RevMan 5.2 or STATA package version 12.0 (Stata Corporation, College Station, Texas, USA).

## 3. Results

### 3.1. Eligible Studies and Characteristics

**Figure 1:**
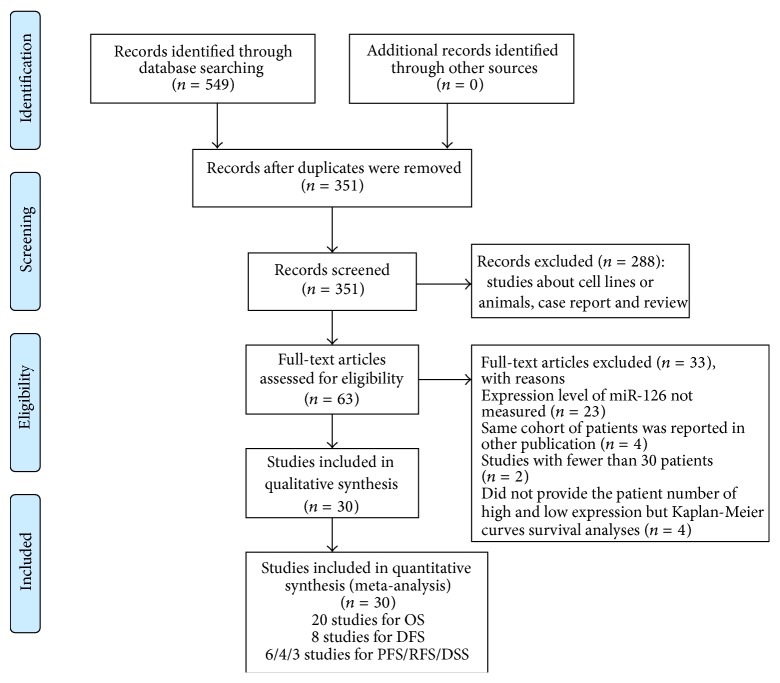
Flow diagram of the study selection process.

A flowchart of detailed searching process is illustrated in Figure 1. Using the described searching strategy above, a total of 549 articles were initially retrieved out of PubMed, Embase, the Cochrane Library, CNKI, and Wan Fang databases. After manually screening the titles, publication types, and abstracts and then checking the full texts by two investigators (Jie Bu and Hui Li), 30 articles were selected for the present meta-analysis [36–65]. Among these eligible studies, 20 studies evaluated the prognostic effect of miR-126 for OS, 8 studies for DFS, and 6/4/3 studies for PFS/RFS/DSS.

The main characteristics and basic information of eligible studies were listed in Table 1 and Table S1 (in Supplementary Material available online at http://dx.doi.org/10.1155/2015/739469). A total of 4497 patients from the United States [63, 65], Spain [53], Japan [36, 37, 57], China [43–48, 51, 52, 58, 62, 64], South Korea [41], Netherlands [38], Norway [40], France [39], Bosnia and Herzegovina [42], Serbia [42], Denmark [49, 50, 54–56], Sweden [55], Canada [61], and Germany [59, 60] were diagnosed with a wide range of carcinomas, including acute myeloid leukemia [36, 38], adult T-cell leukemia [37], non-small cell lung cancer [39–44], colorectal cancer [49, 50, 52, 54–56], laryngeal squamous cell carcinoma [48], esophageal squamous cell cancer [63], hepatocellular carcinoma [45, 46], colon cancer [51, 53], cervical cancer [47], prostate cancer [58], oral cancer [57], breast cancer [59], clear cell renal cell carcinoma [60, 61], esophageal squamous cell carcinoma [62–64], and glioblastoma multiforme [65]. The sample size ranged from 35 to 560. The expression of miR-126 was most often examined in cancerous tissue, while 5 studies examined it in serum/plasma and 1 study tested it in bone marrow. The majority of these studies assessed miR-126 expression by quantitative real-time PCR (qRT-PCR), and in situ hybridization (ISH) was applied in six studies. The most frequently used cut-off value was the median which was applied in 19 studies and the other values were different.

### 3.2. OS Associated with miR-126 Expression

**Figure 2:**
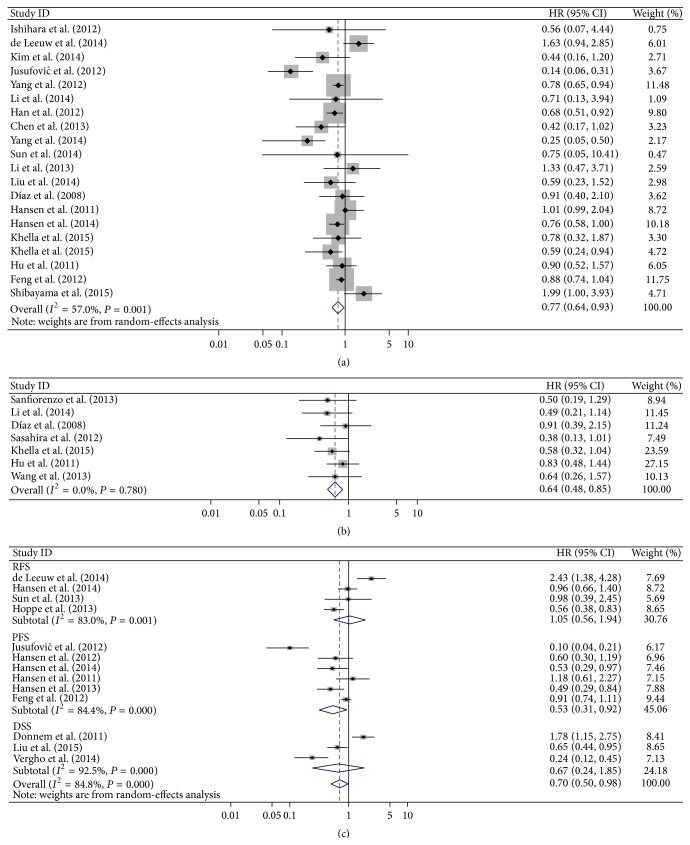
Forest plots of studies evaluating the pooled HR of elevated miR-126 levels for overall survival (OS) (a), disease-free survival (DFS) (b), and recurrence-free survival/progression-free survival/disease-specific survival (PFS/RFS/DSS) (c). Fixed-effects (b) and random-effects (a, c) models were used as the pooling method, respectively.

The main results of this meta-analysis were displayed in Table 2. 20 studies including 3232 cancer patients investigated the relationship between miR-126 expression and the prognosis. For these studies evaluating OS for miR-126, a random-effects model was utilized to calculate the pooled HR and its 95% CI due to the high heterogeneity among these studies (*I* ^2^ = 57.0%, *P* = 0.001). The result showed that high miR-126 level may predict a favorable OS with the combined HR of 0.77 (95% CI: 0.64–0.93; *P*_heterogeneity_ = 0.001) (Table 2, Figure 2(a)).

Furthermore, six subgroup analyses of overall survival were performed which stratified patients by tumor type, ethnicity, sample, assay method, analysis type, and HR estimated (Table 2). Subgroup analyses by tumor type showed that high miR-126 levels were significantly associated with a favorable OS in HCC (HR = 0.65, 95% CI 0.49–0.86, *P*_heterogeneity_ = 0.311). However, AML indicated the opposite result (HR = 1.77, 95% CI 1.15–2.72, *P*_heterogeneity_ = 0.666). In the subgroup analyses by sample type, high miR-126 levels were predictive of better outcome OS in tissue sample (HR = 0.71, 95% CI 0.60–0.85, *P*_heterogeneity_ = 0.01). While elevated miR-126 yielded a worse OS in circulation sample (HR = 1.65, 95% CI 1.09–2.51, *P*_heterogeneity_ *y* = 0.647). With further analyses of studies evaluating OS by ethnicity, we found that the high expression of miR-126 was a significantly favorable predictor for OS in Asians (HR = 0.76, 95% CI 0.66–0.88, *P*_heterogeneity_ = 0.129). Similarly, this conclusion was also found in other subgroups of qRT-PCR assay (HR = 0.72, 95% CI 0.58–0.90, *P*_heterogeneity_ < 0.001), multivariate analysis (HR = 0.81, 95% CI 0.72–0.90, *P*_heterogeneity_ = 0.344), and HRs reported (HR = 0.78, 95% CI 0.64–0.96, *P*_heterogeneity_ ≤ 0.001) (Table 2).

### 3.3. DFS Associated with miR-126 Expression

7 studies included 755 cancer patients evaluated DFS for miR-126, a fixed-effects model was used to assess the pooled effect size due to no heterogeneity among the studies (*I* ^2^ = 0%, *P* = 0.983) (Table 2), and we found that high expression of miR-126 was demonstrated to predict favorable DFS in various cancer (HR = 0.64, 95% CI 0.48–0.85, *P*_heterogeneity_ = 0.780) (Table 2, Figure 2(b)).

Similar to OS analyses, we also performed subtotal investigation for DFS analyses (Table 2). In the subgroup analyses by tumor type, high miR-126 levels were significantly associated with a favorable DFS in NSCLC (HR = 0.49, 95% CI 0.26–0.93, *P*_heterogeneity_ = 0.983). And for ethnicity and analysis type, the high expression of miR-126 was still a significantly better prognosis for DFS (Asian: HR = 0.64, 95% CI 0.44–0.94; *P*_heterogeneity_ = 0.532; Caucasian: HR = 0.63, 95% CI 0.41–0.97, *P*_heterogeneity_ = 0.599; multivariate: HR = 0.65, 95% CI 0.45–0.94; *P*_heterogeneity_ = 0.384; univariate: HR = 0.67, 95% CI 0.50–0.90; *P*_heterogeneity_ < 0.001).

### 3.4. PFS/RFS/DSS Associated with miR-126 Expression

We combined the results for PFS, RFS, and DSS together as PFS/RFS/DSS. A total of 13 studies including 2014 tumor patients focused on PFS/RFS/DSS analysis with significant heterogeneity among them (*I* ^2^ = 67.8%, *P* < 0.001). A random-effects model was applied, and elevated expression of miR-126 was a significant predictor of favorable PFS/RFS/DSS (HR = 0.70, 95% CI 0.50–0.98, *P*_heterogeneity_ = 0.161) (Table 2, Figure 2(c)).

In the subgroup analysis of patients with tumor type, the pooled HR indicated that the high expression of miR-126 was a favorable prognostic marker in CRC (HR = 0.74, 95% CI 0.59–0.94, *P*_heterogeneity_ = 0.108) (Table 2). The same trend was found in subgroup of Asians (HR = 0.69, 95% CI 0.48–0.99, *P*_heterogeneity_ = 0.417) (Table 2).

### 3.5. Heterogeneity Analysis

Obvious heterogeneity of subjects was observed among 13 of the 30 analysis groups, as shown in Table 2. We performed a meta-regression analysis to investigate the sources of this heterogeneity in the OS analysis group (*P* = 0.001, *I* ^2^ = 57%). The obvious heterogeneity was induced by tumor sample (*P* = 0.017) rather than tumor type (*P* = 0.751), miR-126 assay method (*P* = 0.306), patients origin (*P* = 0.631), cut-off values (*P* = 0.772), publication year (*P* = 0.971), and HRs estimate (*P* = 0.836).

### 3.6. Publication Bias and Sensitivity Analysis

**Figure 3:**
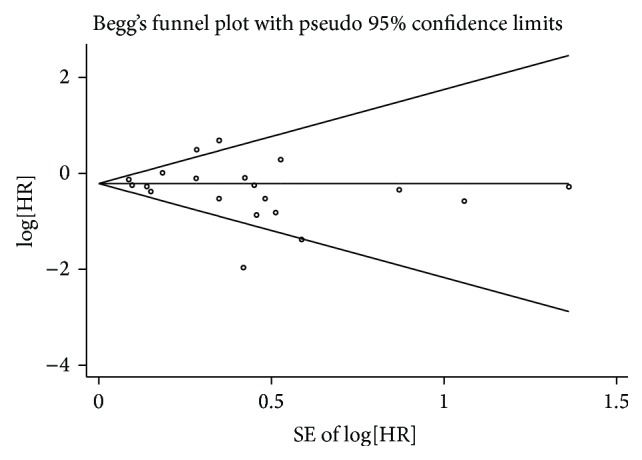
Begg's funnel plots of publication bias test for overall survival (OS).

Begg's funnel plot and Egger's test were used to assess the potential publication bias of the included studies. The funnel plots of the OS, DFS, and PFS/RFS/DSS analysis based on tissue and blood miR-126 did not reveal any evidence of obvious asymmetry. Moreover, the *P* values of Egger's and Begg's tests were all greater than 0.05 in the 30 analysis groups (Table 2, Figure 3, and Figures S1 and S3). Hence, there was no obvious risk of publication bias in our meta-analysis.

**Figure 4:**
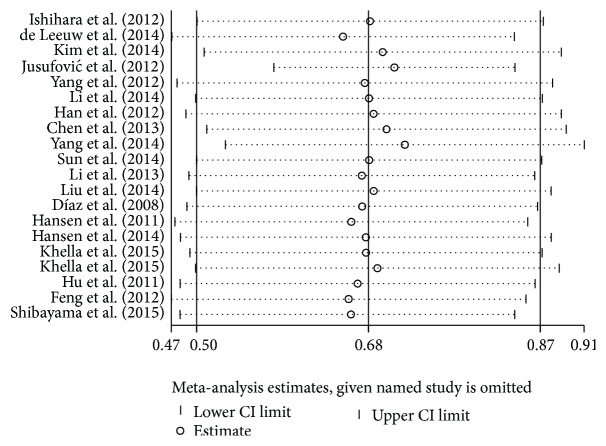
Sensitivity analyses of studies concerning miR-126 and overall survival (OS).

Furthermore, we performed sensitivity analysis to investigate the influence of each individual study on the overall meta-analysis estimate, which computes the pooled HRs by omitting one study in each turn. And there was no obvious influence of individual study on the pooled HRs (Figure 4 and Figures S2 and S4).

## 4. Discussion

Cancer is considered one of the leading causes of death worldwide. The occurrence of cancer is increasing because of the growth and aging of the population, as well as increasing prevalence of established risk factors [66]. Despite the advances in technology and its access, to date, there are few defined prognostic and diagnostic biomarkers available in cancers. Essentially, high cancer mortality rates have remained high, mainly due to the late diagnosis and lack of prognostic markers for various cancers [67]. Hence, many research groups are carrying out studies to develop biomarkers, which can be applied to early detection and correlation of treatment efficacy and prognosis [68].

MiR-126, which is highly expressed in vascular endothelial cells, is one of the most commonly observed cancer-related microRNAs and is dysregulated in most cancers. As one of the major targets of miR-126,* EGFL7* is known to be involved in cell migration and the process of angiogenesis. The conclusion suggests that one of the main functions of miR-126 is to inhibit angiogenesis to reduce blood vessels, which is facilitated by cell migration [69, 70]. Additionally, previous studies have demonstrated that miR-126 may play a role in tumorigenesis and growth by regulating the vascular endothelial growth factor (*VEGF*)/phosphoinositol 3-kinase (*PI3K*)/*AKT* signaling pathways [43, 71]. miR-126 also maintains its role as a suppressor of metastasis that could reduce metastatic rate and size of carcinoma [14, 72]. Furthermore, interactions of miR-126 and* ADAM9* are related to epithelial-mesenchymal transition and the invasive growth of pancreatic cancer cells [73]. In most of the cancers studied, miR-126 functioned as a tumor suppressor and its expression was suppressed; however, several reports using different types of samples have described an oncogenic role for miR-126. Notably, several studies have shown that miR-126 is upregulated in some malignancies due to high tissue specificity, such as gastric cancer, liver cancer, ovarian cancer, and acute myeloid leukaemia [19, 20, 74, 75]. In addition, miR-126 acting as an oncogene, which was found to downregulate* HOXA9*/*PLK*, was often upregulated in myeloid leukaemia and associated with poor prognosis [22, 76]. Moreover, higher expression of miR-126 was shown to be a poor prognostic factor in NSCLC and promote metastasis in prostate cancer [77, 78]. Obviously, it is controversial that miR-126 expression can be used as a prognostic biomarker in different cancers. Hence, in order to evaluate the prognostic role of miR-126 expression in various cancers, we systematically reviewed the published studies and performed a meta-analysis for the first time.

In terms of this, a total of 4497 participants from 30 studies finally were included into the meta-analysis. This result showed that high expression of miR-126 was a significant marker for predicting better outcomes of various cancers (HR was 0.77, 0.64, and 0.70 for OS, DFS, and RFS/PFS/DSS, resp.). For OS, stratified analyses displayed that high expression of miR-126 was a better prognostic marker in HCC, Asians, tissue sample, qRT-PCR assay, multivariate analysis, and HRs reported. However, AML and circulation sample indicated the opposite result. For DFS, subgroup analyses revealed that high expression of miR-126 could predict a favorable DFS in NSCLC, Asian, Caucasian, multivariate, and univariate subgroups. Furthermore, we found that high expression of miR-126 significantly relates to a favorable RFS/PFS/DSS in CRC and Asian subgroup, but no statistical significance is shown in NSCLC, Caucasian, multivariate, and univariate analysis. Additionally, there was no obvious risk of publication bias in our meta-analysis. From the above results, we found that high expression of tissue miR-126 was a positive prognostic factor in cancer patients. But high circulating miR-126 levels predicted a significantly worse OS in patients with cancer. As we know, circulating samples are more convenient to collect and keep monitored, which can effectively evaluate prognosis during or after clinical therapy. Therefore, circulating miR-126 may be an efficacious method for dynamically monitoring the prognosis and therapeutic effects in cancer patients. In this study, only four studies investigated circulating samples, and more studies on these cancers are needed in the future.

Although the present meta-analysis revealed that the expression of miR-126 in cancer patients could be a valuable prognostic biomarker for patients, some limitations should be noticed. Firstly, there was significant heterogeneity existing in our meta-analysis, which was probably attributed to the differences in baseline demographic characters of population, characteristics of patients, the types of cancer, the samples of cancer, the disease stages, the cut-off criteria, the duration of follow-up, and so on. Secondly, several HRs were calculated based on the data extracted from the survival curve; some minor differences exist between the exact HRs and the extrapolated data. Thirdly, due to the lack of a unified cut-off value in miR-126 expression, cut-off values were not consistent among included studies. The different cut-off values may influence the availability of miR-126 as a prognostic biomarker in human cancer. Fourth, in subgroup analyses by sample type and subtype analyses, the number of studies was relatively small. More studies on these cancers are needed in the future. Finally, treatments may influence the expression of miR-126 in cancer samples; however, few researches referred to the treatment effect on HRs or miR-126 expression.

## 5. Conclusion

In sum, in this meta-analysis, we concluded that overexpression of miR-126 was effectively predictive of better prognosis in various carcinomas. Increased miR-126 level in cancerous tissues was associated with favorable OS, DFS, and PFS/RFS/DSS, while elevated circulating miR-126 was indicative of poor OS. However, our results should be regarded cautiously due to the limitations of the present analysis listed above. Further prospective multicenter studies with larger sample size are needed to focus on the relationship between miR-126 and cancer prognosis as well as to explore effective therapies.

## Supplementary Material

S1 PRISMA Checklist. PRISMA 2009 Checklist.
Table S1. HRs and corresponding 95% CIs of eligible studies in the meta-analysis
Figure S1. Begg funnel plots of publication bias test for disease-free survival (DFS).
Figure S2. Sensitivity analyses of studies concerning mir-126 and disease-free survival (DFS).
Figure S3. Begg funnel plots of publication bias test for recurrence free survival/ progression-free survival /disease-specific survival (PFS/RFS/DSS).
Figure S4. Sensitivity analyses of studies concerning mir-126 and recurrence free survival /progression-free survival/disease-specific survival (PFS/RFS/DSS).

## Figures and Tables

**Table 1:** Main characteristics of enrolled studies in the systematic review.

Author	Year	Country	Cancer	Number	Specimen	Assay	Cut-off value	Source of HR	Endpoint	Median follow-up (months)
Shibayama et al. [36]	2015	Japan	AML	108	Bone marrow	qRT-PCR	Median	R	OS	NR
Ishihara et al. [37]	2012	Japan	ATL	35	Plasma	qRT-PCR	Median	SC	OS	NR
de Leeuw et al. [38]	2014	Netherlands	AML	92	Blood	qRT-PCR	Median	R	OS, EFS, RFS	NR
Sanfiorenzo et al. [39]	2013	France	NSCLC	52	Plasma	qRT-PCR	Median	R	DFS	46
Donnem et al. [40]	2011	Norway	NSCLC	332	Tissue	ISH	Expression score ≥ 2	R	DSS^a^	86
Kim et al. [41]	2014	South Korea	NSCLC	72	Tissue	qRT-PCR	Median	R	OS	31
Jusufović et al. [42]	2012	Serbia	NSCLC	50	Tissue	qRT-PCR	Median	R	OS, PFS	5.13
Yang et al. [43]	2012	China	NSCLC	442	Tissue	qRT-PCR	Median	R	OS	24.39–29.28
Li et al. [44]	2014	China	NSCLC	49	Tissue	qRT-PCR	Median	SC	OS, DFS	39
Han et al. [45]	2012	China	HCC	105	Tissue	qRT-PCR	Fold change = 2	R	OS	42.89
Chen et al. [46]	2013	China	HCC	68	Tissue	qRT-PCR	0.70 (ROC curve)	SC	OS	49
Yang et al. [47]	2014	China	Cervical cancer	133	Tissue	qRT-PCR	Median	R	OS	60 (max)
Sun et al. [48]	2014	China	LSCC	38	Plasma	qRT-PCR	Median	SC	OS	NR
Hansen et al. [49]	2012	Denmark	CRC	89	Tissue	ISH	Median	SC	PFS	16.8–26.2
Hansen et al. [50]	2014	Denmark	CRC	63	Plasma	qRT-PCR	Median	R	PFS	8.8–9.2
Li et al. [51]	2013	China	Colon cancer	53	Tissue	ISH	0/1–3+	SC	OS	45.66–55.04
Liu et al. [52]	2014	China	CRC	92	Tissue	qRT-PCR	Median	SC	OS	65
Díaz et al. [53]	2008	Spain	Colon cancer	110	Tissue	qRT-PCR	Median	R	OS, DFS	68
Hansen et al. [54]	2011	Denmark	CRC	81	Tissue	ISH	Median	R	OS, PFS	NR
Hansen et al. [55]	2013	Denmark/Sweden	CRC	89	Tissue	qRT-PCR	Median	R	PFS	NR
Hansen et al. [56]	2015	Denmark	CRC	560	Tissue	qRT-PCR	Median	R	OS, DSS	7 years (max)
Sasahira et al. [57]	2012	Japan	Oral cancer	94	Tissue	qRT-PCR	Means	R	DFS	3.4 years
Sun et al. [58]	2013	China	Prostate cancer	128	Tissue	qRT-PCR	Median	SC	RFS	3–10 years
Hoppe et al. [59]	2013	Germany	Breast cancer	80	Tissue	qRT-PCR	6.20 (ROC curve)	R	RFS	8.84 years
Vergho et al. [60]	2014	Germany	cRCC	37	Tissue	qRT-PCR	3.57 (ROC curve)	R	DSS	41.4
Khella et al. [61]	2015	Canada	cRCC	257,481^b^	Tissue	qRT-PCR	20th percentile	R	OS, DFS, OS^b^	48.6
Liu et al. [62]	2015	China	ESCC	185	Tissue	ISH	Fold change > 3	R	DSS	32
Hu et al. [63]	2011	USA	ESCC	158	Tissue	ISH	1–3+/0–0.5	R	OS, DFS	16.25
Wang et al. [64]	2013	China	ESCC	116	Tissue	qRT-PCR	ΔΔCT < −1	SC	DFS	21–32
Feng et al. [65]	2012	USA	GBM	248	Tissue	qRT-PCR	Median	R	PFS/RFS^b^, OS^b^	NR

CRC: colorectal cancer; HCC: hepatocellular carcinoma; NSCLC: non-small cell lung cancer; cRCC: clear renal cell carcinoma; ESCC: esophageal squamous cell carcinoma; AML: acute myeloid leukemia; ATL: adult T-cell leukemia; LSCC: laryngeal squamous cell carcinoma; GBM: glioblastoma multiforme; qRT-PCR: quantitative real-time PCR; ISH: in situ hybridization; OS: overall survival; DFS: disease-free survival; RFS: recurrence-free survival; PFS: progression-free survival; DSS: disease-specific survival; HR: hazard ratio; SC: survival curve; NR: not reported; R: reported.

^a^DSS included any of the following: DSS, CSS (cancer-specific survival). ^b^Data extracted from TCGA (The Cancer Genome Atlas) in the paper.

**Table 2:** Meta-analysis results.

Outcome	Variables	Number of studies	Number of patients	Model	HR (95% CI)	Heterogeneity		Publication bias	
						*I* ^2^ (%)	*P*	Begg's *P*	Egger's *P*
OS	All	20	3232	Random	0.77 (0.64, 0.93)	56.8	0.001	0.381	0.358
	*Tumor type*								
	NSCLC	4	613	Random	0.42 (0.17, 1.08)	82.2	0.001	1.000	0.340
	HCC	2	173	Fixed	0.65 (0.49, 0.86)	2.60	0.311		
	CRC	5	896	Fixed	0.85 (0.69, 1.04)	0	0.584	0.806	0.679
	RCC	2	738	Fixed	0.65 (0.38, 1.12)	0	0.624		
	AML	2	200	Fixed	1.77 (1.15, 2.72)	0	0.666		
	*Ethnicity*								
	Asian	12	1353	Fixed	0.76 (0.66, 0.88)	37.0	0.129	0.837	0.668
	Caucasian	8	1879	Random	0.77 (0.57, 1.05)	73.8	<0.001	0.536	0.479
	*Sample*								
	Circulation	4	273	Fixed	1.65 (1.09, 2.51)	0	0.647	0.734	0.162
	Tissue	16	2959	Random	0.71 (0.60, 0.85)	51.1	0.01	0.137	0.068
	*Assay method*								
	qRT-PCR	17	2940	Random	0.72 (0.58, 0.90)	61.2	<0.001	0.303	0.250
	ISH	3	292	Fixed	1.00 (0.75, 1.34)	0	0.804	1.000	0.646
	*Analysis type*								
	Multivariate	7	1870	Fixed	0.81 (0.72, 0.90)	11.0	0.344	0.072	0.095
	Univariate	7	1530	Random	0.89 (0.79, 1.00)	66.4	0.007	1.000	0.990
	*HR estimated*								
	HRs reported	14	2897	Random	0.78 (0.64, 0.96)	67.8	<0.001	0.274	0.461
	K-M curve	6	335	Fixed	0.79 (0.53, 1.18)	0	0.666	1.000	0.705

DFS	All	7	755	Fixed	0.64 (0.48, 0.85)	0	0.780	0.133	0.203
	*Tumor type*								
	NSCLC	2	101	Fixed	0.49 (0.26, 0.93)	0	0.983		
	ESCC	2	274	Fixed	0.77 (0.48, 1.24)	0	0.629		
	*Ethnicity*								
	Asian	4	417	Fixed	0.64 (0.44, 0.94)	0	0.532	0.308	0.081
	Caucasian	3	419	Fixed	0.63 (0.41, 0.97)	0	0.599	1.000	0.874
	*Analysis type*								
	Multivariate	3	509	Fixed	0.65 (0.45, 0.94)	0	0.384	0.296	0.360
	Univariate	4	619	Random	0.67 (0.50, 0.90)	88.0	<0.001	0.734	0.586

RFS/PFS/DSS	All	13	2014	Random	0.70 (0.50, 0.98)	84.8	<0.001	0.360	0.288
	*Tumor type*								
	CRC	5	882	Fixed	0.74 (0.59, 0.94)	47.3	0.108	1.000	0.514
	NSCLC	2	382	Random	0.43 (0.03, 7.25)	97.2	<0.001		
	*Ethnicity*								
	Asian	2	313	Fixed	0.69 (0.48, 0.99)	0	0.417		
	Caucasian	11	1701	Random	0.69 (0.46, 1.02)	87.1	<0.001	0.213	0.267
	*Analysis type*								
	Multivariate	7	1531	Random	0.71 (0.50, 1.02)	83.2	<0.001	0.230	0.281
	Univariate	5	651	Random	0.89 (0.77, 1.02)	81.4	<0.001	0.462	0.872

CRC: colorectal cancer; HCC: hepatocellular carcinoma, NSCLC: non-small cell lung cancer; cRCC: clear renal cell carcinoma; ESCC: esophageal squamous cell carcinoma; AML: acute myeloid leukemia; K-M curve: Kaplan-Meier curve; fixed: fixed-effects model; random: random-effects model.
