# Supplementary material for: Prognostic Role of MicroRNA-126 for Survival in Malignant Tumors: A Systematic Review and Meta-Analysis
Source: Dis Markers. 2015 Aug 17;2015:739469. doi: 10.1155/2015/739469 (PMC4553299; doi:10.1155/2015/739469)
Supplement: Supplementary file 1 — S1 PRISMA Checklist. PRISMA 2009 Checklist. Table S1. HRs and corresponding 95% CIs of eligible studies in the meta-analysis Figure S1. Begg funnel plots of publication bias test for disease-free survival (DFS). Figure S2. Sensitivity analyses of studies concerning mir-126 and disease-free survival (DFS). Figure S3. Begg funnel plots of publication bias test for recurrence free survival/ progression-free survival /disease-specific survival (PFS/RFS/DSS). Figure S4. Sensitivity analyses of studies concerning mir-126 and recurrence free survival /progression-free survival/disease-specific survival (PFS/RFS/DSS). [file 739469.f1.docx]

| author | year | country | cancer | Source of HR | endpoint | HR | lower limit | upper limit |
| --- | --- | --- | --- | --- | --- | --- | --- | --- |
| Ishihara K | 2012 | Japan | ATL | SC | OS | 0.56 | 0.07 | 4.44 |
| de Leeuw DC | 2014 | Netherlands | AML | Reported | OS | 1.635(U) | 0.938 | 2.851 |
|  |  |  |  | Reported | RFS | 2.434(U) | 1.385 | 4.279 |
| Sanfiorenzo C | 2013 | France | NSCLC | Reported | DFS | 0.497 | 0.191 | 1.295 |
| Donnem T | 2011 | Norway | NSCLC | Reported | DSS | 1.78(M) | 1.15 | 2.75 |
| Kim MK | 2014 | South Korea | NSCLC | Reported | OS | 0.441(M) | 0.161 | 1.20 |
| Jusufovic E | 2012 | Serbia | NSCLC | Reported | OS | 0.14 | 0.06 | 0.31 |
|  |  |  |  |  | PFS | 0.10 | 0.04 | 0.21 |
| Yang J | 2012 | China | NSCLC | Reported | OS | 0.782(M) | 0.647 | 0.945 |
| Li X | 2014 | China | NSCLC | SC | OS | 0.71 | 0.13 | 3.94 |
|  |  |  |  |  | DFS | 0.49 | 0.21 | 1.14 |
| Han ZB | 2012 | China | HCC | Reported | OS | 0.684(U) | 0.509 | 0.919 |
| Chen H | 2013 | China | HCC | SC | OS | 0.42 | 0.17 | 1.02 |
| Yang Y | 2014 | China | cervical cancer | Reported | OS | 0.252(M) | 0.05 | 0.50 |
| Sun X | 2014 | China | LSCC | SC | OS | 0.75 | 0.05 | 10.41 |
| Hansen TF | 2012 | Denmark | CRC | SC | PFS | 0.60 | 0.30 | 1.19 |
| Hansen TF | 2014 | Denmark | CRC | Reported | PFS | 0.53 | 0.29 | 0.97 |
| Li N | 2013 | China | colon cancer | SC | OS | 1.33 | 0.47 | 3.71 |
| Liu Y | 2014 | China | CRC | SC | OS | 0.59 | 0.23 | 1.52 |
| Diaz R | 2008 | Spain | colon cancer | Reported | OS | 0.91(U) | 0.4 | 2.1 |
|  |  |  |  |  | DFS | 0.91(U) | 0.39 | 2.15 |
| Hansen TF | 2011 | Denmark | CRC | Reported | OS | 1.01 | 0.99 | 2.041 |
|  |  |  |  | Reported | PFS | 1.176 | 0.61 | 2.27 |
| Hansen TF | 2013 | Denmark/Sweden | CRC | Reported | PFS | 0.52(U) | 0.33 | 0.82 |
|  |  |  |  |  |  | 0.49(M) | 0.29 | 0.84 |
| Hansen TF | 2014 | Denmark | CRC | Reported | OS | 0.75(U)  0.76(M) | 0.57  0.58 | 0.98  1.00 |
|  |  |  |  |  | RF-CSS | 0.72(U)  0.96(M) | 0.50  0.66 | 1.04  1.40 |
| Sasahira T | 2012 | Japan | oral cancer | Reported | DFS | 0.247(U)  0.380(M) | 0.09  0.125 | 0.57  1.012 |
| Sun X | 2013 | China | prostate cancer | SC | RFS | 0.98 | 0.39 | 2.45 |
| Hoppe R | 2013 | Germany | Breast cancer | Reported | RFS | 0.560(M) | 0.38 | 0.83 |
| Vergho DC | 2014 | Germany | cRCC | Reported | CSS | 0.50(U)  0.24(M) | 0.28  0.12 | 0.87  0.45 |
| Khella HW | 2015 | Canada | cRCC | Reported | OS | 0.40(U)  0.78(M) | 0.19  0.32 | 0.86  1.87 |
|  |  |  |  |  | DFS | 0.30(U)  0.58(M) | 0.18  0.32 | 0.50  1.04 |
|  |  |  |  | Reported | OS^a^ | 0.59 | 0.24 | 0.94 |
| Liu R | 2014 | China | ESCC | Reported | DSS | 0.622(U)  0.648(M) | 0.422  0.435 | 0.917  0.952 |
| Hu Y | 2011 | USA | ESCC | Reported | OS | 1.37(U)  0.90(M) | 0.89  0.52 | 2.11  1.57 |
|  |  |  |  |  | DFS | 1.37(U)  0.83(M) | 0.89  0.48 | 2.11  1.44 |
| Wang J | 2013 | China | ESCC | SC | DFS | 0.64 | 0.26 | 1.57 |
| Feng J | 2011 | USA | GBM | Reported | PFS^a^ | 0.97(U)  0.91(M) | 0.80  0.74 | 1.17  1.11 |
|  |  |  |  |  | OS^a^ | 0.95(U)  0.88(M) | 0.80  0.74 | 1.13  1.04 |
| Shibayama Y | 2015 | Japan | AML | Reported | OS | 1.958 | 1.001 | 3.927 |

**Table. S1** HRs and corresponding 95% CIs of eligible studies in the meta-analysis. CRC: colorectal cancer, HCC: hepatocellular carcinoma, NSCLC: non-small cell lung cancer, cRCC: clear renal cell carcinoma, ESCC: esophageal squamous cell carcinoma, AML: acute myeloid leukemia, ATL: adult T-cell leukemia, LSCC: laryngeal squamous cell carcinoma, GBM: glioblastoma multiforme, OS: overall survival, DFS: disease-free survival, RFS: recurrence free survival, PFS: progression-free survival, DSS: disease-specific survival, HR: hazard ratio, SC: survival curve, NR: not reported，M: Multivariate, U: Univariate.

^a^data extracted from TCGA( The Cancer Genome Atlas) in the article.



 **Fig. S1** Begg’s funnel plots of publication bias test for disease-free survival (DFS).



 **Fig. S2** Sensitivity analyses of studies concerning mir-126 and disease-free survival (DFS)



 **Fig. S3** Begg’s funnel plots of publication bias test for recurrence free survival/Progression-free survival/disease-specific survival (PFS/RFS/DSS).



 **Fig. S4** Sensitivity analyses of studies concerning mir-126 and recurrence free survival/Progression-free survival/disease-specific survival (PFS/RFS/DSS)
